# A better route to ALPPS: minimally invasive vs open ALPPS

**DOI:** 10.1007/s00464-020-07437-3

**Published:** 2020-04-09

**Authors:** Kawka Michal, Mak Sau, Gall M. H. Tamara, Jiao R. Long

**Affiliations:** HPB Surgical Unit, Dept. of Surgery & Cancer, Imperial College London, Hammersmith Hospital Campus, Du Cane Road, London, W12 0HS UK

**Keywords:** ALPPS, RALPPS, Hepatectomy, Robotic surgery, Laparoscopic surgery, Minimally invasive surgery

## Abstract

**Background:**

Associating liver partition and portal vein ligation for staged hepatectomy (ALPPS) has gained both interest and controversy, as an alternative to portal vein embolisation (PVE) by inducing future liver remnant hypertrophy in patients at risk of liver failure following major hepatectomy. Open ALPPS induces more extensive hypertrophy in a shorter timespan than PVE; however, it is also associated with higher complication rates and mortality. Minimally invasive surgery (MIS), with its known benefits, has been applied to ALPPS in the hope of reducing the surgical insult and improving functional recovery time while preserving the extensive FLR hypertrophy.

**Methods:**

A search of the PubMed, Medline, EMBASE and Cochrane Library databases was conducted on 10 July 2019. 1231 studies were identified and screened. 19 open ALPPS studies, 3 MIS ALPPS and 1 study reporting on both were included in the analysis.

**Results:**

1088 open and 46 MIS-ALPPS cases were included in the analysis. There were significant differences in the baseline characteristic: open ALPPS patients had a more diverse profile of underlying pathologies (*p* = 0.028) and comparatively more right extended hepatectomies (*p* = 0.006) as compared to right hepatectomy and left extended hepatectomy performed. Operative parameters (time and blood loss) did not differ between the two groups. MIS ALPPS had a lower rate of severe Clavien–Dindo complications (≥ IIIa) following stage 1 (*p* = 0.063) and significantly lower median mortality (0.00% vs 8.45%) (*p* = 0.007) compared to open ALPPS.

**Conclusion:**

Although MIS ALPPS would seem to be better than open ALPPS with reduced morbidity and mortality rates, there is still limited evidence on MIS ALPPS. There is a need for a higher quality of evidence on MIS ALPPS vs. open ALPPS to answer whether MIS ALPPS can replace open ALPPS.

Bi-lobar or large unilobar liver tumours pose a surgical challenge. Resection with a clear margin (R0) gives patients the best chance for long-term survival, but often can only be achieved by removing a large amount of liver parenchyma, leaving patients with an insufficient future liver remnant liver volume (FLRV). It is generally agreed that FLRV should to be at least 25–30% of the total liver volume (TLV) [1–3] to avoid post-hepatectomy liver failure (PHLF), the main cause of postoperative mortality amongst this group of patients [4]. Compromised liver function associated with liver cirrhosis, neoadjuvant chemotherapy, severe fatty liver or obstructive jaundice increases the minimal required FLRV to up to 40% of the TLV [5]. The need for extensive complete resection but also preserving a sufficient FLR leaves only 10–29% of patients suitable for this surgery at presentation [6–8]. The current paradigm for patients with insufficient FLR is to induce hypertrophy before hepatectomy, the gold standard of which is portal vein embolisation (PVE), which can increase FLRV by 11.9% to 38% in 4–8 weeks [9, 10], ultimately leading to increased respectability. However, one of the problems associated with PVE is the failure to progress to stage 2 hepatectomy in 15–20% of patients due to insufficient FLRV hypertrophy or micro- and macrometastatic spread of the disease occurring between two stages of the procedure [9, 10]. An alternative method of achieving FLR hypertrophy has been sought and thus the conceptualisation of associating liver partition and portal vein ligation for staged hepatectomy (ALPPS).

Since the first case series on ALPPS published by Schitzbauer et al. [11] in 2012, it has gained vast interest but also controversy. Classic ALPPS is a modification of two-stage hepatectomy; stage 1 being an open operation to remove any malignant lesions from the FLR (usually the left lobe) combined with the in-situ splitting of the liver parenchyma and right portal vein ligation [11]. The trauma to liver tissue caused by parenchymal transection and the subsequent inflammatory response (elevated IL-6 and TNF-alpha) is thought to be responsible for producing a more profound FLR hypertrophy in ALPPS than PVE [12]. Imaging is then used to confirm sufficient FLR hypertrophy before proceeding to stage 2, which involves, most commonly, a right or a right extended hepatectomy [11]. Initial case series of ALPPS reported not only a more extensive FLR hypertrophy but also the process occurring in a shorter timespan (74% FLRV increase in 9 days) compared to PVE. However, mortality and morbidity rates (12% and 64%, respectively) are higher than that seen with PVE (1.5% mortality and 14% morbidity) [10, 11]. Further studies confirmed classic ALPPS as a procedure with considerable morbidity (ranging from 33 to 58%) which remains higher than PVE [13–15]. One of the most common complications in the early days of ALPPS was bile leak after in-situ splitting, leading to infection, sepsis and death [16]. It was also suggested that hypertrophy achieved in a short timespan might not translate to functional gain, in which case the procedure would not benefit patients and subject them to additional risks compared to PVE [17].

Since the conception of the initial ALPPS technique, multiple modifications have been introduced to address some of the inherent limitations of the original method. Variations to the ‘in-situ splitting’ part of the procedure such as partial ALPPS, mini-ALPPS, tourniquet ALPPS (ALTPS), and hybrid ALPPS as well as the application of different energy devices for parenchymal splitting [microwave ablation-assisted ALPPS (LAPS) and radiofrequency ablation-assisted ALPPS (RALPPS)] [16, 18–22] have been reported in recent years. Minimising the impact of stage 1 by avoiding a ‘true’ in-situ split and performing a virtual or partial parenchyma splitting is the common theme of the modified techniques, as such change allows for faster patient recovery prior to stage 2, as well as eliminating some of the complications in stage 1 and reducing blood loss. ALPPS variants were suggested to be potentially associated with a significant reduction in morbidity and mortality rates when comparing with classic ALPPS [23], although the quality and heterogeneity of evidence did not allow to reach the conclusion on whether a variant of ALPPS is superior to conventional ALPPS or whether ALPPS could replace PVE [24]. Two RCTs have been published on ALPPS vs PVE [14] and RALPPS vs PVE [25], respectively. The initial results of those trials are encouraging; more experience, refining of the technique and introducing modifications to original technique result in high FLR hypertrophy rate (68 to 80%) but can reduce the morbidity to a level comparable with PVE [25].

Another route of improvement of ALPPS in line with paradigm of reducing the impact of two open surgeries in quick succession is the application of both laparoscopic and more recently robotic surgery to RALPPS and ALPPS in the hope that the recognised benefits of minimally invasive liver surgery (MIS) would reduce the morbidity and mortality from ALPPS, improving patient outcomes [25–31]. This review aims to pool the available evidence in MIS ALPPS and compare it with the more established open ALPPS.

## Methods

### Search strategy

A systematic review of the existing literature was conducted. Medline, EMBASE, PubMed and Cochrane Libraries databases were included and searched. Boolean search terms: ‘ALPPS’, ‘Associating liver partition for portal vein ligation for staged hepatectomy’ and ‘in-situ split’ were used, with no restriction on publishing date. Identified abstracts were then reviewed independently by two authors (MK and SM) and discrepancies were reviewed by a third author (TMHG). Whenever a study was not available, authors were contacted to obtain the missing studies. Article abstracts were manually screened for any missing studies. The last search was conducted on 10 July 2019.

### Inclusion criteria

Case series of minimum 20 cases were included in open ALPPS group for analysis. After the same criterion for study size was applied to MIS-ALPPS studies, only 1 study satisfying it was found, and hence it was decided that a limit of minimum five cases would be applied to MIS-ALPPS group.

All the studies that included ALPPS and one or more other interventions, in which the data regarding ALPPS were presented in a separate and extractable manner, were included. Moreover, if a study reported multiple subgroups (differentiated according to tumour type, surgical technique, modification of ALPPS, etc.), all subgroups with a sufficient number of cases were included in the analysis. If there was evidence that two studies included the same patients (e.g. subsequent studies by the same research group), the study reporting a larger total number of cases was chosen. As far as studies using data from the ALPPS registry are concerned, only one study with the biggest sample size reporting outcomes of interest to this review was included to avoid repetition of cases.

### Exclusion criteria

Conference abstracts, case reports, animal studies and studies not reported in English were excluded from the analysis. Studies, in which ALPPS was used as a salvage procedure for failed PVE/other procedures, were excluded from the analysis.

### Data extraction

Data extraction was conducted separately by two authors (MK and SM). Severe complications, according to Clavien–Dindo classification (≥ IIIa), were analysed. Evidence quality of the studies was assessed according to Oxford Centre for Evidence-based Medicine [32].

### Outcomes

Baseline patient characteristics including age, sex, tumour type, neoadjuvant chemotherapy, sFLRV before stage 1 were extracted. Operative data regarding both stages including the surgical technique in both stages, length of surgery, estimated blood loss, RBC transfusions, R0 margin were extracted. Type of hepatectomy was classified according to the Brisbane terminology [33].

The data regarding the interval between stage 1 and stage 2 (sFLRV before stage 2, the interval between stages and %FLR hypertrophy) were collected. Finally, the information regarding the postoperative course including total length of hospital stay and CD complication following both stages and 90 day mortality was collected.

### Statistical analysis

Whenever outcomes were reported as median and interquartile range it was estimated into mean ± SD for the purpose of analysis using the method described by Hozo et al. [34]. Parametric data were analysed using Students *t* test, while non-parametric data were analysed using a Mann–Whitney *U* test for continuous variables. Categorical data were analysed using the *χ*^2^ test. The alpha value of less than 0.05 was considered significant. RevMan 5.0 and Minitab 19.0 software and were used for analysis.

## Results

**Fig. 1:**
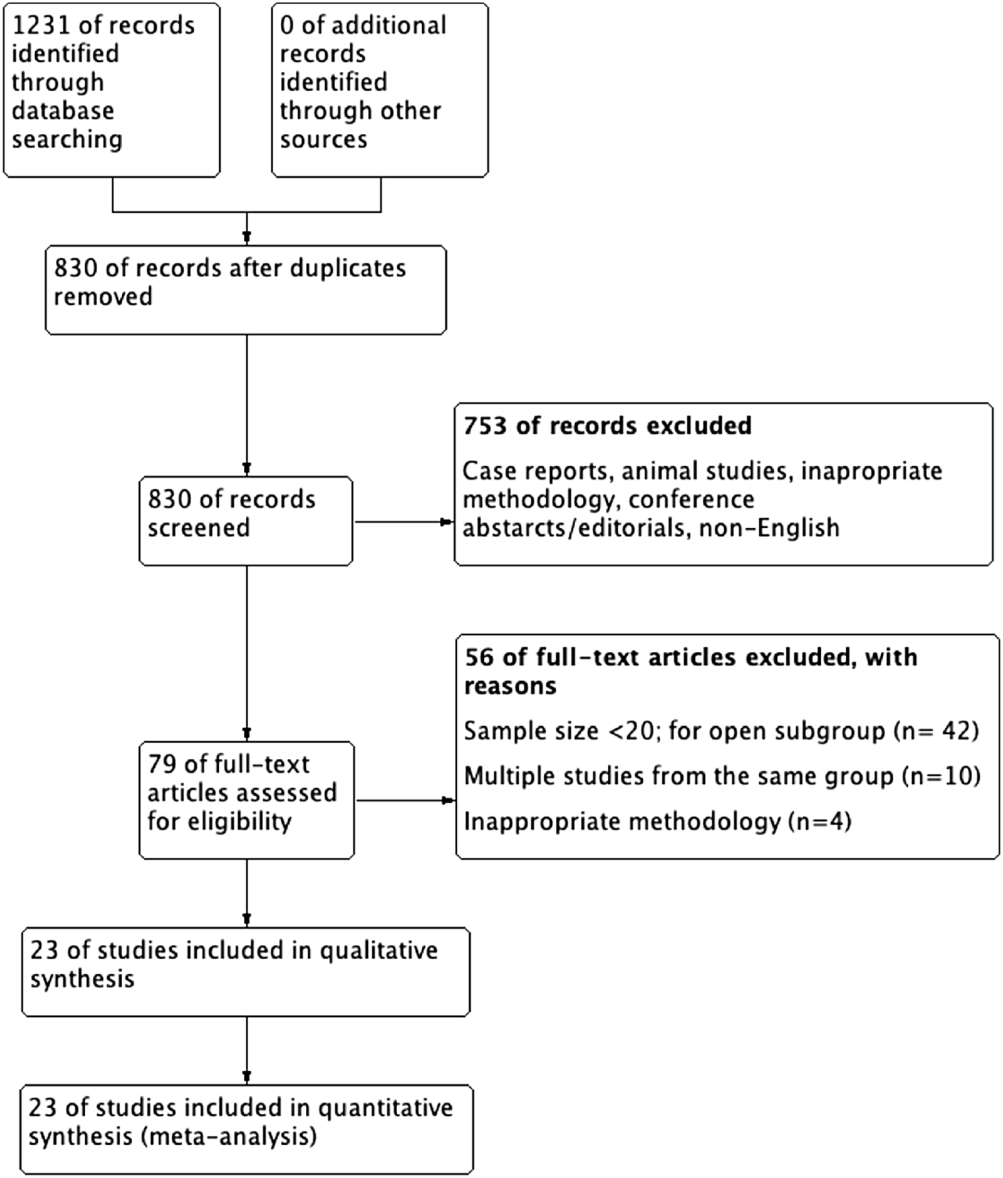
Search strategy and reasons for exclusion of studies. Out of 23 studies, 3 reported on MIS ALPPS and 19 reported on open ALPPS studies while 1 study reported on both

We identified 3 MIS-ALPPS studies, 19 open ALPPS studies and 1 comparative study reporting on both open and MIS ALPPS (Fig. 1). After studies including more than one subgroup were accounted for, 24 open ALPPS and 4 MIS-ALPPS groups were established for comparison. Since not all the studies reported all the outcomes of interest or reported them in a form that did not yield itself to the analysis of choice, specific outcome comparisons are conducted with the exclusion of said studies. MIS studies and open studies are presented in Table 1. Classic ALPPS technique was described in detail in the introduction. Partial ALPPS includes 50–80% degree of parenchymal transection instead of full in-situ splitting. Tourniquet-ALPPS avoids actual transection of the liver and instead utilises a tourniquet tied on the future transection plane. Mini-ALPPS variant includes partial parenchymal transection, but additionally aims to avoid liver mobilisation and hilar dissection. Hybrid ALPPS variant separates ALPPS procedure into 3 steps instead of regular 2: in-situ splitting, followed by PVE using intervention radiology and final hepatectomy after FLR hypertrophy is achieved.

**Table 1:** Reported open and MIS ALPPS and variants

Study	Evidence quality	ALPPS	
		Stage 1 technique	Stage 2
MIS ALPPS (*n* = 46)			
Gall et al. [22]	3b	Laparoscopic RALPPS (*n* = 4) Open RALPPS (*n* = 1)	R hepatectomy (*n* = 5) (5/5 open)
Machado et al.* [35]	3b	Laparoscopic ALPPS (*n* = 10)	R hepatectomy (*n* = 3) Extended R hepatectomy (*n* = 7) (10/10 laparoscopic)
Truant et al. [36]	4	Laparoscopic mini-ALPPS (*n* = 5)	Extended R hepatectomy (*n* = 5) (5/5 open)
Jiao et al. [25]	1b	Laparoscopic RALPPS (*n* = 24) Robotic RALPPS (*n* = 2)	R hepatectomy (*n* = 19) Extended R hepatectomy (*n* = 5) Stage 2 not completed (*n* = 2) (13 open, 10 laparoscopic, 1 robotic)
Open ALPPS (*n* = 1088)			
Schnitzbauer et al. [11]	4	Classic ALPPS (*n* = 25)	Extended R hepatectomy (*n* = 25)
Shindoh et al. [37]	3b	Classic ALPPS (*n* = 25)	Extended R hepatectomy (*n* = 25)
Torres et al. [38]	4	Classic ALPPS (*n* = 39) Laparoscopic ALPPS (*n* = 2/39)	Extended R hepatectomy (*n* = 37) Stage 2 not completed (*n* = 2)
Schadde et al. [39]	3b	Classic ALPPS (*n* = 48)	Extended R hepatectomy (*n* = 48)
Alvarez et al. [40]	4	Classic ALPPS (*n* = 9) Partial ALPPS (*n* = 21) Laparoscopic ALPPS (*n* = 1/30)	R hepatectomy (*n* = 8) Extended R hepatectomy (*n* = 20) Extended L hepatectomy (*n* = 1) Stage 2 not completed (*n* = 1)
Rosok et al. [41]	4	Classic ALPPS (*n* = 36)	R hepatectomy (*n* = 6) Extended R hepatectomy (*n* = 29) Extended L hepatectomy (*n* = 1)
Truant et al. [42]	4	Classic ALPPS (*n* = 62) Laparoscopic ALPPS (*n* = 2/62)	R hepatectomy (*n* = 31) Extended R hepatectomy (*n* = 28) Stage 2 not completed (*n* = 3)
Kambakamba et al. [43]	3b	Classic ALPPS (*n* = 38)	R hepatectomy (*n* = 13) Extended R hepatectomy (*n* = 23) Stage 2 not completed (*n* = 2)
Linecker et al. [44]	3b	Partial ALPPS (*n* = 22)	R hepatectomy (*n* = 4) Extended R hepatectomy (*n* = 18)
		Classic ALPPS (*n* = 23)	R hepatectomy (*n* = 7) Extended R hepatectomy (*n* = 15) Stage 2 not completed (*n* = 1)
Serenari et al. [45]	4	Classic ALPPS (*n* = 22)	R hepatectomy (*n* = 12) Extended R hepatectomy (*n* = 10)
		Classic ALPPS (*n* = 20)	R hepatectomy (*n* = 3) Extended R hepatectomy (*n* = 17)
Chan et al. [46]	4	Classic ALPPS (*n* = 13) Partial ALPPS (*n* = 12)	R hepatectomy(*n* = 25)
Machado et al. [35]	3b	Classic ALPPS (*n* = 20)	R hepatectomy (*n* = 5) Extended R hepatectomy (*n* = 13) Stage 2 not completed (*n* = 2)
Wanis et al. [47]	4	Classic ALPPS (*n* = 47)	R hepatectomy (*n* = 12) Extended R hepatectomy (*n* = 32) Extended L hepatectomy (*n* = 3)
Sandstrom et al. [14]	1b	Classic ALPPS (*n* = 48)	R hepatectomy (*n* = 25) Extended R hepatectomy (*n* = 19) Stage 2 not completed (*n* = 4)
Serenari et al. [48]	4	Classic ALPPS (*n* = 7) Partial ALPPS (*n* = 15) Mini-ALPPS (*n* = 4)	R hepatectomy (*n* = 10) Extended R hepatectomy (*n* = 15) Extended L hepatectomy (*n* = 1)
Schnitzbauer et al. [49]	2c	Classic ALPPS (*n* = 196) Partial ALPPS (*n* = 12) Hybrid ALPPS (*n* = 4) Tourniquet ALPPS (*n* = 8)	Extended R hepatectomy (*n* = 220)
		Classic ALPPS (*n* = 154) Partial ALPPS (*n* = 14) Hybrid ALPPS (*n* = 3) Tourniquet ALPPS (*n* = 12)	R hepatectomy (*n* = 183)
Wang et al. [50]	4	Classic ALPPS (*n* = 45)	R hepatectomy (*n* = 4) Extended R hepatectomy (*n* = 37) Stage 2 not completed (*n* = 4)
Robles-Campos et al. [51]	3b	Tourniquet ALPPS (*n* = 34) Laparoscopic (*n* = 4/34)	R hepatectomy (*n* = 23) Extended R hepatectomy (*n* = 11)
Torzilli et al. [52]	3b	Classic ALPPS (*n* = 26)	R hepatectomy (*n* = 11) Extended R hepatectomy (*n* = 13) Stage 2 not completed (*n* = 2)
Vennarecci et al. [53]	4	Classic ALPPS (*n* = 10) Partial ALPPS (*n* = 14)	R hepatectomy (*n* = 20) Extended R hepatectomy (*n* = 4)

### Demographic data

There was no significant difference in age or sex between the open and MIS-ALPPS groups (Table 2). When the underlying pathology was compared, open ALPPS group included a significantly different distribution of tumour types (*p* = 0.028). The biggest difference stemmed from the fraction of CRLM (80.61% in open vs 97.83% in MIS). No cases of HCC and CCA were reported in the MIS-ALPPS group. The use of neoadjuvant chemotherapy was significantly more in MIS-ALPPS group (87.80% vs. 70.80%, *p* = 0.028). Stage 1 technique also significantly differed (*p* < 0.001), as only 14.43% of open ALPPS cases were performed using any modification of ALPPS, compared to all the cases in MIS-ALPPS group. When technique at stage 2 was compared, there was a significant difference in distribution (*p* = 0.006) as right hepatectomy was more commonly performed following a MIS ALPPS (61.36% vs 36.02%) and right extended hepatectomy was more frequent following open ALPPS (62.92% vs 38.64%).

**Table 2:** Demographic data of the pooled cohorts of MIS ALPPS and open ALPPS. Data reported as the number and (%) unless stated otherwise

	Open (n = 1088)	Minimally invasive (*n* = 46)	*p* value
Age (median ± IQR)	58.70 (57.00–62.13)	60.25 (50.75–61.99)	0.800
Male (%)	720 (66.4)	25 (55.4)	0.083
Neoadjuvant chemotherapy (%)	725 (70.8)	36 (87.8)	0.017
Type of tumour (%)			0.028
CRLM	877 (80.61)	45 (97.83)	
HCC	105 (9.65)	0 (0.00)	
CCA	56 (5.15)	0 (0.00)	
Other*	50 (4.60)	1 (2.17)	
Type of ALPPS (%)			< 0.001
Classic ALPPS	931 (85.57)	0 (0.00)	
Modified ALPPS**	157 (14.43)	46 (100)	
Type of hepatectomy (%)			0.006
Right hepatectomy	402 (37.68)	27 (61.36)	
Right extended hepatectomy	659 (61.76)	17 (38.64)	
Left extended hepatectomy	6 (0.56)	0 (0.00)	

*Other tumour types include non-colorectal liver metastases, sarcomas, neuroendocrine tumours and other not specified tumours

**Modified ALPPS include any modification to the classic technique described by Schnitzbauer et al. [11] (energy source, type of parenchyma splitting, etc.)

### Interval between stage 1 and stage 2

The median interval between stages of ALPPS (Table 3) was shorter in open ALPPS group (10.25 vs. 20.13 days) than in MIS-ALPPS group; however, the difference was not significant (*p* = 0.136). Similarly, open ALPPS resulted in a higher % FLR hypertrophy (81.00% vs. 74.49%) but there was no statistical significant difference (*p* = 0.823). When failure to progress to stage 2 was compared, the median dropout rate was 0% in MIS ALPPS and 1.5% in the open ALPPS group (*p* = 0.447).

**Table 3:** Data regarding the interval between stage 1 and stage 2 of ALPPS

	Open ALPPS	MIS ALPPS	*p* value
Time between stages (days) (Open = 586, MIS = 46)	10.25 (7.44–14.45)	20.13 (10.85–21.41)	0.136
FLR hypertrophy (%) (Open = 921, MIS = 46)	81.00 (68.50–90.50)	74.49 (63.33–101.74)	0.823
Dropout rate before stage 2 (%) (Open = 660, MIS = 46)	1.50 (0.00–7.10)	0.00 (0.00–5.78)	0.447

Data presented as median (IQR)

### Operative data

Median stage 1 operating (Table 4) time was shorter by 61.12 min in MIS-ALPPS groups (211.88 vs 273.00 min) but longer by 15.5 min during stage 2 (200.00 vs 184.50 min). In both differences were not significant (*p* = 0.249 and 0.498, respectively). When estimated blood loss was compared in MIS-ALPPS group, it was lower during stage 1 (275.00 vs 494.00 ml, *p* = 0.362) but higher during stage 2 (650.00 vs 305.00 ml, *p* = 0.110). Both differences in blood loss were not statistically significant.

**Table 4:** Intraoperative data regarding stage 1 and stage 2 of ALPPS

	Open ALPPS	MIS ALPPS	*p* value
Stage 1 length (min) (Open = 721, MIS = 46)	273.00 (186.94–315.56)	211.88 (121.56–285.88)	0.249
Stage 2 length (min) (Open = 318, MIS = 41)	184.50 (142.50–216.63)	200.00 (196.50–215.00)	0.498
Stage 1 blood loss (ml) (Open = 269, MIS = 41)	494.00 (272.50–675.00)	275.00 (202.50–315.00)	0.362
Stage 2 blood loss (ml) (Open = 269, MIS = 41)	305.00 (162.50–400.00)	650.00 (387.50–962.50)	0.110

Data presented as median (IQR)

### Postoperative data

Morbidity and mortality data are shown in Table 5. There were no significant differences in stage 1, stage 2 and combined severe CD complications. Stage 1 complications were lower in MIS ALPPS (median 0% vs. 11%, *p* = 0.063). Stage 1 bile leak rate was also lower in MIS-ALPPS group (0% vs 4.7%, *p* = 0.291) but no statistical significance was found. All MIS ALPS studies reported 0% post-stage 2 liver failure, which did not allow for statistical analysis; the rates of liver failure in open ALPPS group were 13.60% (median). The total length of hospital stay was 4.2 days shorter in MIS-ALPPS group (15.30 vs. 19.50) but there was no significant difference (*p* = 0.108). 90-day mortality was significantly lower in the MIS-ALPPS group (median 0% vs 8.45%, *p* = 0.007).

**Table 5:** Postoperative data regarding stage 1 and stage 2 of ALPPS

	Open ALPPS	MIS ALPPS	*p* value
Stage 1 CD > IIIa (%) (Open = 684, MIS = 36)	11.00 (4.55–22.85)	0.00 (0.00–3.80)	0.063
Stage 2 CD > IIIa (%) (Open = 720, MIS = 36)	14.40 (11.00–24.25)	15.40 (0.00–40.00)	0.933
Combined CD > IIIa (%) (Open = 305, MIS = 20)	36.00 (15.78–43.78)	0.00 (0.00–40.00)	0.175
Stage 1 bile leak (%) (Open = 806, MIS = 41)	4.70 (0.00–21.00)	0.00 (0.00–15.00)	0.291
Post-stage 2 liver failure (%) (Open = 970, MIS = 46)	13.60 (5.08–26.70)	0.00 (0.00–0.00)	N/A
Total length of hospital stay (days) (Open = 336, MIS = 41)	19.50 (17.19–24.50)	15.30 (12.50–18.50)	0.108
90 days mortality (%) (Open = 1041, MIS = 46)	8.45 (5.68–12.00)	0.00 (0.00–2.85)	0.007

Data presented as median (IQR)

*CD* Clavien–Dindo score

## Discussion

This pooled analysis of the observational studies compares the results of open ALPPS and MIS ALPPS (laparoscopic and robotic). Our results, albeit based on small sample size, show that application of minimally invasive surgical methods to the original ALPPS protocol is feasible and safe and may eliminate some of the original technique’s limitations.

### Demographic data

The majority of cases in MIS-ALPPS group are CRLM, which is an important consideration, as a previous risk analysis study has reported that patients with CRLM have lower levels of severe Clavien–Dindo complications and lower mortality after ALPPS compared to other types of malignancies [15]. Although not represented in the studies chosen for MIS-ALPPS groups in this analysis, both HCC and different types of CCA have been previously successfully operated on using MIS ALPPS [54, 55]. What is more, for hilar cholangiocarcinoma the precision provided by the endowrist of the robot can provide an advantage, as hilar dissection can be a technically challenging part [55, 56].

The distribution of pathologies is also further reflected in the significant difference of neoadjuvant chemotherapy administration as its use in the treatment of CRLM is much more common than in the treatment of HCC [57, 58]. Interestingly, right extended hepatectomies more often followed open ALPPS, while right hepatectomies followed the majority of MIS ALPPS. This difference might be attributed to tumour location, size, number of lesions and their distribution (tumour-related data were reported inconsistently, and hence not included in the analysis), as well as a limited sample size of MIS-ALPPS subgroup, which might not be reflective of the procedure as whole. It is important to acknowledge that right extended hepatectomy (as compared to right hepatectomy) is associated with higher morbidity and mortality as compared to right hepatectomy ALPPS [49]. The volumetric data were insufficient to compare the sFLR size before stage 1 and stage 2 between open and MIS-ALPPS groups.

### The interval between stage 1 and stage 2 data

There still is no consensus on what the optimal interval between the stages of ALPPS, as in the analysed studies the median interval ranges from 7 to 28 days. In the MIS-ALPPS group, the interval was nearly double (20.13 vs 10.25 days) that of open ALPPS. The extended interval might be beneficial for the patients, as it allows for the hypertrophy to be coupled by the hyperplasia of the cells, which would provide additional functional gains and help to avoid PHLF. In our analysis, hypertrophy using MIS ALPPS and open ALPPS was comparable with no statistically significant difference between two values.

A possible benefit of using MIS during state one is the reduction of adhesions, which allows for stage 2 to be performed without the urgency. Fibrous adhesions can be a hindrance in case of stage 2 of open ALPPS, increasing the time and blood loss of the procedure [59, 60] [61, 62]. The mechanism of hypertrophy is not well understood; however, there is evidence which supports the theory that hypertrophy is due to the systemic rather than solely local response [23]. Studies have also reported that well-defined partial (> 50% of parenchyma thickness) or virtual transection (radiofrequency or microwave ablation) produce comparable FLR hypertrophy to full in-situ split, while possibly lowering the morbidity of stage 1 [18, 44]. The hypertrophy following ALPPS might also be related to lack of shunting and collateralization of circulation after step 1 [63].

### Operative data

Operation duration exceeding 300 min and RBC transfusions are independent risk factors for ALPPS, hence the analysis of the operative parameters [4, 15]. We have found no significant difference between both stage 1 and stage 2 operative time and blood loss. There were insufficient data regarding RBC transfusions in MIS-ALPPS group studies to compare this operative parameter. This shows that while laparoscopic and robotic ALPPS can be technically challenging with less freedom of movement, they can perform non-inferiorly to open ALPPS in this regard, despite additional time needed for equipment setup.

### Postoperative course

When compared to PVE, open ALPPS is often criticised due to its comparatively high morbidity and mortality. One of the proposed solutions to this problem is using minimally invasive surgery (MIS), which has known benefits in reducing morbidity, length of hospital stay and allowing for better functional recovery of the patients. We have not found a significant difference in severe CD complications (> IIIa) both in stage 1 and stage 2; however, stage 1 severe complications were lower in MIS-ALPPS groups (median 0% vs 11%, *p* = 0.063). Since bile leak following stage 1 and post-stage 2 liver failure are two of the most common complications in ALPPS, those parameters were analysed separately; there was no difference in bile leak and liver failure rates could not be analysed. It is worth noting that studies reported liver failure according to different criteria (50/50, ISGLS, etc.) so the comparison might not be fully reflective of true rates of liver insufficiency and failure. The total length of hospital stay was also lower in MIS ALPS group by 4.2 days, which is associated with better recovery by the patients. If stage 1 is performed laparoscopically/robotically, then the patient can leave the hospital in the interval before stage 2, which can reduce the possible complications associated with prolonged hospital stay (which might be needed in case of open stage 1 followed by quick stage 2). There is still not enough evidence to establish how such difference would translate to patient functional recovery. We have also collected data regarding resection margin and the % of R0 resections; however, only 1 of the 4 MIS-ALPPS studies reported this outcome (75% of R0 resection compared to average 90.4% of R0 in pooled open ALPPS studies). The final variable analysed was 90-day mortality, which was found to be significantly lower in MIS-ALPPS group (*p* = 0.007).

### Advantages of MIS ALPPS

We believe that performing ALPPS and its variants using MIS approach can be a greatly beneficial modification to classic ALPPS procedure, helping to bring it more in line with the gold standard of liver hypertrophy inducing procedures—PVE. PVE is a less invasive procedure and hence it generates fewer complications than an extensive procedure such open ALPPS stage 1. Despite no statistically significant found, the stage 1 severe CD complications and total hospital length of stay were lower in MIS-ALPPS group compared to the open group, which is promising for when more minimally invasive ALPPS data are generated and more robust comparisons can be conducted. As far as robotic ALPPS is concerned, on top of the aforementioned precision provided by the endowrist, some other benefits include better control of intrahepatic vasculature without the need of full liver mobilisation. Endosuturing in the case of intraoperative bleeding is also facilitated by the robotic arms [64]. High-definition 3D vision provided by the robotic camera provides a high-quality image, which is valuable for precision of dissection and manoeuvring in limited spaces [56].

### Disadvantages of MIS ALPPS

Despite promising initial results, both laparoscopic and robotic ALPPS are technically demanding procedures and should be performed by experienced HPB surgeons with adequate laparoscopic/robotic training and experience in ALPPS. The inability to identify lesions by palpation during surgery can also be a limitation; accurate mapping of tumours combined with intraoperative ultrasound scan is required for successful MIS-ALPPS procedure [35]. Additional training required, as well as the scarcity of robots, limits the use of robotic ALPPS to high-volume HPB centres equipped with the required technology. Furthermore, the implementation costs of robotic surgery systems, although reduced since its introduction, act as an obstacle to robotic systems being universally embraced, in light of no long-term patient outcome data to support it as of now [65, 66].

### Limitations

One of the major limitations of this study is the lack of evidence to support minimally invasive ALPPS, as currently there is just one study with more than 20 cases in ALPPS group and only 4 case series in total. This limits the viability of comparisons of open ALPPS with MIS ALPPS, as the sample size (1088 vs 46) is unequal, which translates to less powerful statistical tests. One of the possibilities to overcome the problem of unequal sample sizes, would be 1:1 case matching, based on the preoperative patients’ characteristics and surgery type; however, due to the poor quality of reported data and heterogeneity of reporting, such methodology could not have been adopted.

In the open ALPPS group, 9/1088 cases were performed laparoscopically; however, they were not reported separately, and hence they could not have been excluded and could potentially confound the results obtained. The MIS-ALPPS groups due to its small size might not be representative of the technique as a whole. What is more, since there were only 4 studies in MIS-ALPPS groups, some of the outcomes of interest could not be analysed as they were not reported in the sufficient number of studies (R0, sFLR, RBC transfusions). Moreover, the evidence level of studies included in both groups is low (only 2 RCTs) which is a major hindrance in reaching widely applicable conclusions.

Secondly, there are also significant discrepancies in underlying pathology and choice of procedure between the groups, showing that open ALPPS can currently be used on a much wider and varied population as compared to MIS ALPPS. Subgroup analysis was not possible due to small sample size in the MIS-ALPPS groups. While the technical feasibility of MIS ALPPS on tumour types other than CRLM has been shown, there are still insufficient data to assess minimally invasive ALPPS on other pathologies. In light of such heterogeneity of the two groups, the results of this study can only be applied to the CRLM subgroup of ALPPS patients. Due to the insufficient data on MIS ALPPS, the results of this study can only be treated as preliminary, yet promising findings. Thirdly, due to the novelty of both the techniques (an especially MIS ALPPS), there is a lack of long-term patient follow-up (and when present reported in a heterogenous manner that does not yield itself to analysis) for both MIS and open ALPPS, which makes it hard to comprehensively assess the benefits of such surgery for patients.

### Conclusion

Minimally invasive surgery and its associated benefits offer an alternative to open ALPPS procedure and can potentially improve peri- and postoperative outcomes compared to the classic variant of the technique. There is still a need for more minimally invasive ALPPS data in order to draw meaningful conclusions, but the preliminary analysis of the available case series shows potential in reducing morbidity, mortality and length of hospital stay, all of which can help transform ALPPS into a less invasive technique, aligning it closer with the gold standard PVE procedure while allowing for extensive FLR hypertrophy.
